# Impacts of hesperidin on whey protein functionality: Interacting mechanism, antioxidant capacity, and emulsion stabilizing effects

**DOI:** 10.3389/fnut.2022.1043095

**Published:** 2023-01-04

**Authors:** Yin Wang, Yangkai Guo, Longtao Zhang, Meilan Yuan, Li Zhao, Chunqing Bai, David Julian McClements

**Affiliations:** ^1^National R&D Branch Center for Freshwater Fish Processing, College of Life Science, Jiangxi Science and Technology Normal University, Nanchang, China; ^2^College of Food Science, Fujian Agriculture and Forestry University, Fuzhou, Fujian, China; ^3^Department of Food Science, University of Massachusetts, Amherst, MA, United States

**Keywords:** whey protein, hesperidin, interaction, molecular docking technology, emulsions

## Abstract

The objective of this work was to explore the possibility of improving the antioxidant capacity and application of whey protein (WP) through non-covalent interactions with hesperidin (HES), a citrus polyphenol with nutraceutical activity. The interaction mechanism was elucidated using several spectroscopic methods and molecular docking analysis. The antioxidant capacity of the WP-HES complexes was analyzed and compared to that of the proteins alone. Moreover, the resistance of oil-in-water emulsions formulated using the WP-HES complexes as antioxidant emulsifiers to changes in environmental conditions (pH, ion strength, and oxidant) was evaluated. Our results showed that HES was incorporated into a single hydrophobic cavity in the WP molecule, where it was mainly held by hydrophobic attractive forces. As a result, the microenvironments of the non-polar tyrosine and tryptophan residues in the protein molecules were altered after complexation. Moreover, the α-helix and β-sheet regions in the protein decreased after complexation, while the β-turn and random regions increased. The antioxidant capacity of the WP-HES complexes was greater than that of the proteins alone. Non-radiative energy transfer from WP to HES was detected during complex formation. Compared to WP alone, the WP-HES complexes produced emulsions with smaller mean droplet diameters, exhibited higher pH and salt stability, and had better oxidative stability. The magnitude of these effects increased as the HES concentration was increased. This research would supply valuable information on the nature of the interactions between WP and HES. Moreover, it may lead to the creation of dual-function antioxidant emulsifiers for application in emulsified food products.

## 1. Introduction

Whey protein (WP), a co-product of cheese making, is widely used in the food industry as an emulsifying, foaming, thickening, film-forming, and encapsulating agent (1). The excellent nutritional profile and diverse functional attributes of WP have led to its widespread application a broad spectrum of food products (2–7). Like many other foods proteins, however, the application of WP is often limited because of its tendency to aggregate when exposed to certain types of environmental conditions, such as pH values around the isoelectric point, high salt levels, and heating above the thermal denaturation temperature. Moreover, WP only has a relatively low ability to retard lipid oxidation, which limits its application as a natural antioxidant in foods. The functional properties of proteins can often be improved by modifying their structures (8). Physical, chemical, and biological modification methods have been developed and employed for this purpose. Compared to other methods, physical modifications are often more affordable and environmentally friendly, and do not require regulatory approval (6, 8–12). Recently, improving the antioxidant activity of food proteins by physically attaching natural antioxidant molecules to their surfaces has gained increasing popularity.

Phenolic compounds are usually secondary metabolites produced by plants, which have at least one aromatic ring with one or more hydroxyl groups attached (5, 13). These molecules can scavenge free radicals and prevent oxidative damage, thereby acting as natural antioxidants. They have also been reported to exhibit various other health-promoting effects, including antitumor, anti-inflammatory, and antimicrobial activities (14). Phenolic compounds can be combined with proteins to create protein-phenolic complexes that can be used as multi-functional food ingredients, such as antioxidant emulsifiers. Numerous studies have reported that phenolic compounds can spontaneously bind to proteins *via* non-covalent interactions, and alter their structures and functionalities (8, 15).

Various kinds of proteins (e.g., whey protein, casein, and gelatin) and polyphenols (e.g., tannic acid, curcumin, and phenolic acids) have been shown to form protein-polyphenol complexes with different functional properties (15–18). For example, chlorogenic acid was reported to bind to casein and WP through non-covalent interactions, which altered the structure, solubility, and foaming capacity of the proteins (19). Proanthocyanidins have been reported to interact with rice proteins, which altered the secondary structure and surface hydrophobicity of the proteins (20). Other researchers reported that tyrosol and hydroxytyrosol in olive oil extracts showed no or weak binding to several food proteins (caseinate, bovine serum albumin, β-lactoglobulin, and gelatin), whereas other olive oil phenolics showed strong binding (21). Overall, these studies show that the strength of the binding interaction, the conformational changes of the proteins, and the functionality of the complexes formed depend on polyphenol and protein type (5, 22).

In this study, we examined interactions between WP and hesperidin (HES). HES is a flavonoid glycoside phenolic compound, commonly found in citrus fruits such as oranges and lemons (23, 24). Because of its potential health benefits such as antihyperlipidemic, cardioprotective, antihypertensive, antidiabetic, and antioxidant activities, HES has attracted considerable attention for its potential application as a nutraceutical ingredient in foods (25, 26). Previous studies have reported the interaction of HES with various proteins, including glutenin (23), HMG-CoA reductase enzyme (27), SARS-CoV2 spike protein (28) and digestive enzyme (29). The results of these researches suggest that HES exerts strong binding affinity to these proteins. Jiang et al. (23) also reported that HES could improve the functional properties (thermal properties and emulsifying activity) of edible protein. It is known that the interacting mechanism varies greatly depending on protein and phenolic type (13, 14). For instance, previous studies suggest that the binding of HES to glutenin (23) was dominated by hydrogen bonding and hydrophobic forces, whereas the binding of HES to α-amylase was dominated by van der Waals forces and hydrogen bonding (29). Consequently, it is important to establish the binding mechanism for specific protein-phenolic pairs, as well as the impact of these interactions on the functionality of the complexes formed.

Whey proteins are commonly used as emulsifiers in the food industry to produce nutraceutical-loaded oil-in-water emulsions or nanoemulsions (30, 31). Typically, the WPs are dissolved in water and then homogenized with an oil phase containing the nutraceutical. The protein molecules adsorb to the oil-water interface and form a protective coating around the oil droplets, which can protect the nutraceuticals from chemical degradation, e.g., oxidation. However, WP only has limited potential to inhibit lipid oxidation in emulsions. We hypothesized that complexing WP with HES would improve its emulsifying and antioxidant properties, thereby leading to the formation of complexes that could be used as dual-purpose antioxidant emulsifiers in foods. Consequently, we used a combination experimental measurements and molecular dynamics simulations to understanding the interactions between WP and HES and their impact on protein functionality.

## 2. Materials and methods

### 2.1. Materials and reagents

WP (purity > 90%) was purchased from Shanghai Yuanye Biotech Co., Ltd., Diphenyl-2-picrylhydrazyl free radical (DPPH), HES, KBr (spectral purity), and 2,4,6-tris(2-pyridyl)-s-triazine (TPTZ) were purchased from Aladdin Industrial Company (Shanghai, China). All other chemical reagents were of analytical grade. Double distilled water was used throughout the research. WP powder was dissolved in phosphate buffer (PB) solution (10 mM, pH 6.80) to obtain a stock WP solution of 0.5 mg/mL. This solution was diluted to 0.1 mg/mL with PB before use. An aqueous HES solution was prepared by weighing HES powder into water, adjusted the pH 12 using 0.1 mol/L NaOH for sufficiently soluble, and then made up to a constant volume using distilled water to obtain a stock solution containing 1 × 10^–3^ mol/L of HES.

### 2.2. Fluorescence spectra

A series of complex solutions with final HES concentrations from 0 to 26.7 μM was prepared according to Li et al. (32). Briefly, increasing aliquots of HES solution was dropped into 3.0 mL of 0.1 mg/mL WP. The mixture was adjusted to pH 6.80 with 0.01 M/L hydrochloric acid solution, equilibrated for 30 min, and then measured on a Model F-7000 fluorescence spectrofluorometer (Hitachi, Tokyo, Japan). The fluorescence emission spectra of the mixture samples kept at 298, 304, and 310 K, respectively, were recorded at an excitation wavelength of 280 nm.

### 2.3. Synchronous fluorescence spectrometry

Synchronous fluorescence spectrometry of the mixed samples was carried out using the same method described in section “2.2 Fluorescence spectra” but Δλ values of 15 and 60 nm were used to monitor the changes in the local environment of the tyrosine and tryptophan residues in the WP, respectively.

### 2.4. Ultraviolet- visible (UV-VIS) spectra measurements

Ultraviolet- visible (UV-VIS) spectra of samples placed in cuvettes were collected from 300 to 450 nm using a UV-visible spectrophotometer (UV-1800, Shanghai Meipuda Instrument Co., Ltd., Shanghai, China).

### 2.5. Fourier transform infrared spectroscopy (FTIR)

Fourier transform infrared spectroscopy (FTIR) spectra of freeze-dried samples mixed with potassium bromide (1:100 w/w) were recorded over the wavenumber range from 400 to 4,000 cm^–1^. Amide I band (1,600–1,700 cm^–1^) of the spectra was deconvoluted using Peak-Fit v4.12 software (SPSS Inc., Chicago, IL). The secondary structures of the protein were then analyzed as the percentages of α-helixes, β-sheets, β-turns, and random coils.

### 2.6. Molecular docking simulation

The model structure of HES (ID: 10621) was downloaded from PubChem database.^1^ The model structures of α-Lactalbumin (α-LA) and β-lactoglobulin (β-LG) named as PDB ID: 1F6S and 1BEB, respectively, were downloaded from the RCSB database^2^ and then used to carry out the molecular docking simulations, since α-LA and β-LG are the most abundant constituents in WP. Before being imported into the AutoDock Vina Program (AutoDock 1.5.6), the original water molecules and ligands in the α-LA and β-LG 3D structure were deleted using PyMOL tools. In addition, hydrogen atoms and charges were added to the protein molecule structures prior to carrying out the simulations. HES and α-LA/β-LG were set as the ligand and receptor, respectively. The interaction between HES and α-LA/β-LG was then modeled using the AutoDock Vina Program. The docking was performed taking the center of the HES as the grid center (β-LG: −4.5, 4, 16. α-LA: 35, 50, 10) and docking boxes of size was 50Å × 40 Å × 55Å (β-LG) and 120Å × 160 Å × 72Å (α-LA), respectively. Top 10 poses of the docking results were saved, and the one that showed the lowest interaction energy was regarded as the optimized result. The interactions between HES and α-LA/β-LG were visualized using the Discovery Studio 2016 program.

### 2.7. Determination of antioxidant activity

The influence of HES binding on the antioxidant capacity of WP was evaluated by DPPH assay and ferric reducing power (FRAP assay).

#### 2.7.1. DPPH assay

The DPPH radical scavenging capacity of the samples was determined using a method described previously (33), with some modifications. 1 mL of the test sample was mixed with 2 mL of DPPH reagent and then stored in the dark at room temperature for 30 min. The absorbance of the mixtures was then measured at 517 nm using a UV spectrophotometer (UV-1800, Shanghai Meipuda Instrument Co., Ltd., Shanghai, China). Results were expressed as the Trolox equivalent (TE) antioxidant capacity (mg of TE/L of sample).

#### 2.7.2. FRAP assay

The ferric reducing power of the samples was determined according to a method described previously (34), with slight modifications. Briefly, 1 mL of sample was added to 4.5 mL of FRAP working reagent, mixed thoroughly, and then placed in the dark at 37°C. After reaction for 10 min, the absorbance of the samples was measured at 593 nm using UV-1800 UV spectrophotometer. The ferric reducing power of HES-WP complexes, free HES, and free WP were measured and compared. Results are expressed in terms of the TE antioxidant capacity.

### 2.8. Stability WP-HES based emulsion

#### 2.8.1. Nanoemulsion preparation

A 1.0 wt% WP solution was prepared by dispersing powdered WP (1.0 g) into 100 mL of 10 mM PB solution (PBS, pH 6.8) and stirring at ambient temperature for 5 h. The resulting solution was then stored overnight at 4°C and filtered (Fisher Scientific, PA) to remove any insoluble particles before further use. HES stock solutions were obtained by dispersing powdered HES powders in water as described in section “2.1 Materials and reagents” Different volumes of HES stock solution were added into the WP solution, sonicated for 15 min, and then stirred for 2 min to obtain HES-WP complexes with final HES concentrations of 1 or 2 mM. Coarse emulsions were formed by blending 90 wt% aqueous solutions containing WP or HED-WP complexes with 10 wt% coix seed oil at 12,000 rpm for 2 min by using a high-speed mixer. To fabricated fine emulsions, these coarse emulsions were then homogenized on a high-pressure homogenizer for 5 times at 110 MPa. Sodium azide (0.02 wt%) was added to prevent bacterial growth (please note, this is a non-food grade antimicrobial agent).

#### 2.8.2. Particle size and zeta potential measurement

The particle size distribution and zeta potential of the emulsions were determined using a laser diffraction particle size analyzer (LS 12 320, Beckman Coulter, Inc., Brea, CA). To avoid multiple scattering, samples were diluted 100-fold by using PBS (pH 6.8, 10 mM) before measurement.

#### 2.8.3. Morphological characterization

The microstructure of the emulsions was imaged using an inverted fluorescence microscope (ECLIPSE Ti-U, Nikon Co., Japan). Before analysis, 200 μL of emulsion was thoroughly mixed with an equal volume of PBS. Then, 20 μL of Nile red solution (1 mg/ml) were added into the mixture and the system was shaken to dye the oil phase. An aliquot of sample was then dropped on to a microscope slide, covered by a cover slip, and then imaged by using a 60 × oil objective lens.

#### 2.8.4. The pH stability of emulsions

Emulsions made with WP or HED-WP complexes were adjusted to different pH values (pH 3.0–9.0) by adding a small amount of HCl (1 M) or NaOH (1 M) solutions. All samples were placed in 10 ml capped test tubes and stored at 4°C overnight. The particle size distribution, zeta potential, and microstructure of the emulsions were then determined.

#### 2.8.5. Salt-stability

The salt-stability of emulsions prepared using either WP or HED-WP complexes as an emulsifier was determined by measuring their particle size distribution, zeta potential, and microstructure after they being exposed to a range of NaCl solutions (0–500 mM) overnight.

#### 2.8.6. Oxidization stability

Freshly prepared coix oil loaded emulsions were placed in glass tubes with screwed caps, and then incubated in the dark for 15 days at 55°C. Samples were withdrawn periodically during storage for analysis of the extent of lipid oxidation. The primary reaction production (peroxide value, PV) and secondary reaction product (thiobarbituric acid reactive substances, TBARS) in the emulsions systems were determined according to the methods reported (35, 36). The content of lipid PV was calculated based on a standard curve of cumene hydroperoxide and expressed as mmol hydroperoxide per kg oil. The TBARS content was calculated from a standard curve prepared using 1, 1, 3, 3-tetramethoxypropane, and expressed as mmol malondialdehyde equivalent per kg oil.

### 2.9. Statistical analysis

All experiments were carried out at least three times and the data were expressed as the means ± standard deviation (SD). Significant differences were analyzed on “SPSS 19.0” statistical software using one-way ANOVA with Duncan’s test, and *p* < 0.05 was considered as statistically significant.

## 3. Results and discussion

### 3.1. Fluorescence emission spectrum

**FIGURE 1:**
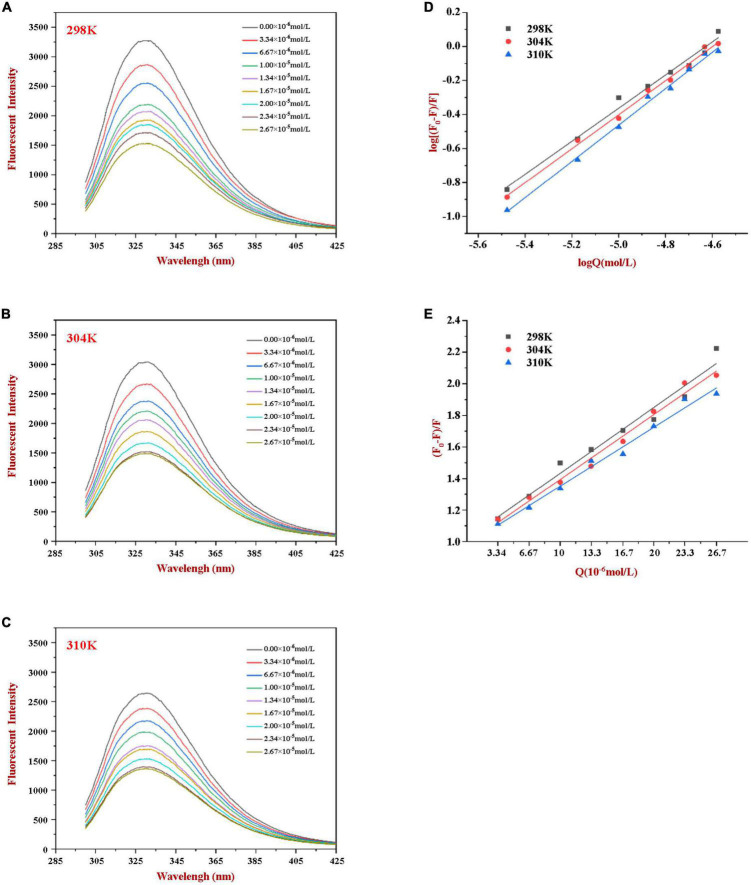
Fluorescence emission profiles of WP at various concentrations of HES (0–26.7 μM) at 298K **(A)**, 304K **(B)**, and 310K **(C)**. The Double-log plots **(D)** and Stern-Volmer plots **(E)** of the interaction between WP and HES at different temperatures.

Phenylalanine, tyrosine, and tryptophan are the main fluorescent amino acids in proteins. Of these, tryptophan is the dominant intrinsic fluorophore. It exhibits intrinsic protein fluorescence when excited at 280 nm and observed at around 350 nm (20). The intensity and wavelength of the resulting intrinsic protein fluorescence peak is highly sensitive to the local environment of the tryptophan residues (22). In our experiment, the fluorescence emission spectra of WP in the presence of various concentrations of HES were measured at different temperatures to explore the interactions between HES and WP. As shown in Figure 1, a strong fluorescence emission peak at approximately 345 nm was observed. The fluorescence intensity decreased gradually as the HES content was increased. Moreover, a slight red shift of about 2 nm occurred in the wavelength where the peak maximum was observed when the HES levels were increased. The observed reduction in the fluorescence peak intensity, and shift in the peak position, of WP in the presence of HES is indicative of a binding interaction between the protein and polyphenol molecules (13, 22). The red shift suggests that the microenvironment of the tryptophan residues was altered in a manner that was consistent with a decrease of hydrophobicity or increase in polarity in the binding sites in the protein molecules (5). Similar results have been reported by other researchers. Dai et al. found that resveratrol could quench rice glutelin’s intrinsic fluorescence and alter its maximum peak of 2 nm (20) Kong et al. reported the ability of xylitol to quench the intrinsic fluorescence intensity of WP in a dose dependent-manner (5).

### 3.2. Fluorescence quenching mechanism

According to the literature, fluorescence quenching mechanisms can be categorized as either dynamic mechanism or static mechanism (8, 23). To establish the mechanism between WP and HES, the fluorescence quenching behavior of the protein and polyphenol was measured at three different temperatures (298, 304, and 310 K) and the data were then analyzed using a Stern-Volmer Eq. (1). As shown in Figure 1, the Stern-Volmer curves exhibited good linearity (*R* > 0.99) for all temperatures used (Figure 1E), indicating that quenching was either due to a collisional or a static mechanism. Table 1 shows that the K_*SV*_ values decreased gradually as the temperature increased, being 4.44 × 10^4^, 3.92 × 10^4^, and 3.43 × 10^4^ L⋅mol^–1^ for 298, 304, and 310 K, respectively. Moreover, the smallest K_*Q*_ value (343 × 10^10^ L⋅mol^–1^⋅s^–1^) was much higher than 2.0 × 10^10^ L⋅mol^–1^⋅s^–1^ (the maximum diffusion collision quenching constant), indicating that HES could induce static quenching of WP. In addition, the decrease in K_*SV*_ values with increasing temperature suggests the interaction complex formed was less stable at higher temperatures (22).

**TABLE 1:** Quenching constants K_*SV*_, binding constants Ka, and the number of binding sites *n* for the interaction between WP and HES at different temperatures.

T (K)	K_*SV*_ (× 10^4^L⋅mol^–1^)	K_*a*_ (× 10^5^L⋅mol^–1^)	*n*	K_*Q*_ (× 10^11^L⋅mol^–1^⋅s^–1^)	*R*
298	4.44 ± 0.25^a^	7.54 ± 0.02^a^	0.9791 ± 0.04^c^	44.4 ± 0.25^a^	0.993
304	3.92 ± 0.11^b^	3.89 ± 0.11^b^	0.9984 ± 0.03^b^	39.2 ± 0.11^b^	0.997
310	3.43 ± 0.18^c^	3.14 ± 0.05^c^	1.0745 ± 0.02^a^	34.3 ± 0.18^c^	0.998

R is the correlation coefficient for the K*_SV_* values. Results are expressed as means ± *SD* (*n* = 3). Values with different uppercase letters in the same column are significantly different (*p* < 0.05).

For static quenching, Eq. (2) was used to calculate the binding constant (K_*a*_) and the number (n) of binding site of the WP and HES interaction. As summarized in Table 1, the n values were all approximately equal to 1, suggesting that WP had one binding site for HES. The calculated K_*a*_ values of the WP-HES complexes decreased as the temperature increased, indicating that the binding force was diminished at higher temperatures, which is in consistence with K_*SV*_ (23). The weaker affinity of HES to WP may be due to the decrease in the strength of the hydrophobic interactions when the temperature was raised (5, 32).


(1) $$\frac{{\text{F}}_{0}}{\text{F}}=1+{\text{K}}_{\text{Q}}\tau_{0}\left[\text{Q}\right]=1+{\text{K}}_{\text{SV}}\left[\text{Q}\right]$$

(2) $$\log\frac{{\text{F}}_{0}-\text{F}}{\text{F}}=\log{\text{K}}_{\text{a}}+\text{n}\log\left[\text{Q}\right]$$

Here, F_0_ and F represent the fluorescent intensity of WP before and after HES was added, respectively. Also, τ0 (10^–8^ s) is the fluorescence lifetime in the absence of quenchers, [Q] is the concentration of HES, K_*SV*_ is quenching constant for Stern-Volmer, K_*Q*_ is the quenching rate parameter, K_*a*_ is binding constant, and n is the number of binding sites.

### 3.3. Thermodynamic parameters

To better understand the main forces contributing to the formation of the WP-HES complexes, the enthalpy change (ΔH), entropy change (ΔS), and free energy change (ΔG) were calculated using the Van’t Hoff equation Eq. (3) followed by the free energy Eq. (4)


(3) $${\text{InK}}_{\text{a}}=-\frac{\Delta \text{H}}{\text{RT}}+\frac{\Delta \text{S}}{\text{R}}$$

(4) ΔG=ΔH-TΔS

Here, R is the gas constant (8.314 J⋅mol^–1^K^–1^), T is the absolute temperature (298, 304, or 310 K), and K_*a*_ is the binding constant, which is the same one shown in Eq. (2). The ΔH and ΔS values were obtained from the slope and intercept of the linear Van’t Hoff curves based on ln K_*a*_ vs. 1/T, respectively, then ΔG was calculated using Eq. (4).

Four types of binding forces (hydrophobic, hydrogen bonding, van der Waals, and electrostatic forces) have been reported to play a role in the formation of non-covalent protein-polyphenol complexes (37). Insights into the relative importance of these forces can often be obtained by examining the relative signs of the free energy, enthalpy, and entropy changes. Negative ΔG values indicate spontaneous binding; positive ΔH and negative ΔS indicates electrostatic and hydrophobic interactions are the predominant force; negative ΔH and ΔS suggest van der Waals forces or hydrogen bonds play a major role in the interaction; positive ΔH and ΔS involve hydrophobic forces are the main force; negative ΔH and positive ΔS values indicate electrostatic forces are dominate (20). As shown in Table 2, ΔG was below zero, indicating that the binding of HES with WP was spontaneous. Meanwhile, the positive ΔS and ΔH (0.27 kJ⋅mol^–1^⋅K^–1^ and 55.0 kJ⋅mol^–1^) suggesting that hydrophobic forces are dominate driving forces for the WP and HES interaction. This result is consistent with previous studies, which also reported that hydrophobic interactions were the main driving force involved in formatting soy protein-resveratrol complexes (37, 38). WP-xylitol complex (5), and rice glutelin-procyanidin complex (37).

**TABLE 2:** Thermodynamic parameters for the interaction of WP with HES at different temperatures.

T (K)	*ΔG* (kJ⋅mol^–1^)	*ΔS* (kJ⋅kmol^–1^⋅K^–1^)	*ΔH* (kJ⋅mol^–1^)
298	-25.43 ± 0.22^a^	0.27 ± 0.55	55.03 ± 0.46
304	-27.05 ± 0.13^b^		
310	-28.67 ± 0.09^c^		

Results are expressed as means ± *SD* (*n* = 3). Values with different uppercase letters in the same column are significantly different (*p* < 0.05).

### 3.4. Non-radiative energy transfer

Based on Förster theory, non-radiative energy transfer usually occurs when there is sufficient overlap of the fluorescence emission spectrum of the donor molecule and the ultraviolet absorption spectrum of the acceptor molecule, and the distance between the two molecules is below 7 nm (39). Energy transfer rate, spectral overlap, and distance between the donor and recipient can be calculated using the following equations:


(5) $$\text{E}=1-\frac{\text{F}}{{\text{F}}_{0}}=\frac{{\text{R}}_{0}^{6}}{{\text{R}}_{0}^{6}+{\text{r}}_{6}}$$

(6) $${\text{R}}_{0}^{6}=8.8\times 10^{-25}{\text{K}}^{2}{\text{n}}^{-4}\emptyset \text{J}$$

(7) $$J=\frac{\sum F\left(\lambda \right)\varepsilon \left(\lambda \right){\text{λ}}^{4}\Delta \lambda }{\sum F\left(\lambda \right)\Delta \lambda }$$

Here, F_0_ and F are the same values as in Eq. (1), E is the rate of energy transfer, r is the Förster distance between the WP and HES, R_0_ is the critical energy transfer when 50% energy is transferred, K^2^ is the dipole orientation factor, n is the refractive index of the medium, J is the spectral overlap integral, Ø is the fluorescence quantum yield of the WP in the absence of the HES, F(λ) and e(λ) are the WP’s corrected fluorescence intensity and the HES’s molar extinction coefficient at the wavelength of λ, respectively.

**FIGURE 2:**
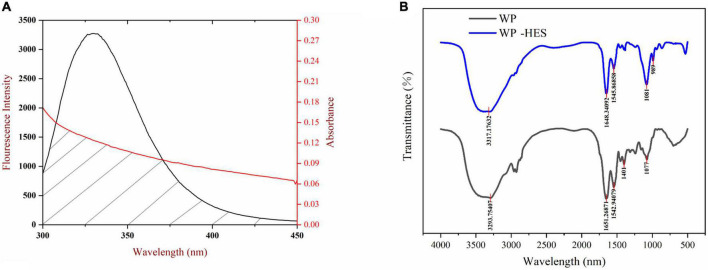
The overlapping area of the UV-VIS absorption spectrum of HES and the WP fluorescence spectrum **(A)**, the red line and black line represent UV-VIS absorption spectrum and fluorescence spectrum, respectively; FTIR spectrograms of WP and WP-HES **(B)**.

In our experiment, WP and HES are assumed to be the donor and acceptor, respectively, and the values of n, K^2^, and Ø were taken to be 1.336, 2/3, and 0.12, respectively. The spectral overlap of the WP emission spectrum and the HES absorption spectrum is shown in Figure 2. Based on the above equations, E = 0.126, *r* = 2.896 nm, and *R*_0_ = 2.097 nm. The value of distance “r” is smaller than 7 nm and within the range of 0.5 R_0_ and 1.5 R_0_, which suggests that the energy transfer occurs (from WP to HES), resulting in static quenching (29).

### 3.5. Synchronous fluorescence

**FIGURE 3:**
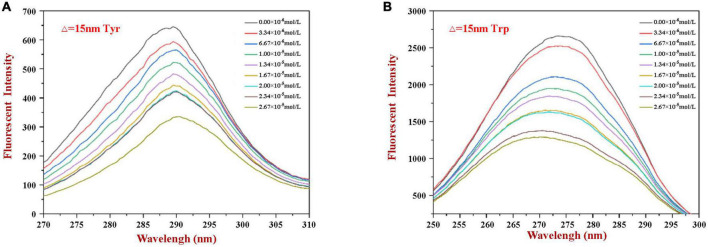
The synchronous fluorescence spectrum of WP in the absence and presence of HES at Δλ = 15 nm **(A)** and Δλ = 60 nm **(B)**.

Synchronous fluorescence spectroscopy is useful for providing information about changes in the local microenvironment of several amino acid residues in proteins, which can be used to analyze the influence of ligand binding on protein conformation (29, 32). When the Δλ values are set at 15 and 60 nm, this method can provide characteristic information about changes in the microenvironments of tyrosine and tryptophan residues, respectively (5). Figure 3 shows the synchronous fluorescence spectra of WP in the presence of various concentrations of HES. Obviously, the fluorescence intensity of tryptophan (at maximum peak) was markedly higher than that of tyrosine (at maximum peak), indicating that tryptophan was the main contributor to the WP fluorescence signals. Moreover, the fluorescence intensity of the two residues decreased gradually with increasing HES concentration. When the HES concentration was 2.67 × 10^–5^ mm/ml, there was a decrease of 48.7 and 56.8% in the original fluorescence intensities of the tyrosine and tryptophan, respectively. The higher reduction in tryptophan fluorescence intensity may be because it was much closer to the binding sites than tyrosine, and therefore contributed more to the binding process (40). Taken together, the fluorescence and synchronous fluorescence spectroscopy measurements indicate that WP and HES formed non-fluorescent complexes (19). Meanwhile, both maximum peaks appeared to show slight shifts with increasing HES concentration. When Δλ was set at 60 nm, a blue shift from 275 to 270 nm occurred, which indicated that the hydrophobicity of the tryptophan microenvironment increased and/or its polarity decreased after HES bound to the WP (5, 38). This effect is consistent with the non-polar HES molecules binding to the hydrophobic pocket on the surface of the WP molecules where the tryptophan residues are located, thereby resulting in an increase in the hydrophobicity of their microenvironment (37). When Δλ was set at 15 nm, a small red shift from 290 to 292 nm was observed, suggesting only a slight decrease in hydrophobicity and/or increase in polarity around the tyrosine residues. These changes may be due to the fact that more tyrosine residues were exposed to an aqueous environment after binding, which may have been due to a change in protein conformation (19, 27). The observed microenvironment changes of the tyrosine and tryptophan residues therefore suggest there was a change in the conformation of the WP molecule conformation after HES binding.

### 3.6. FTIR spectroscopy

Fourier transform infrared spectroscopy spectroscopy can be used to provide information about changes in the secondary structure of proteins (41). Several characteristic peaks were obtained for WP, which could be related to the presence of specific functional groups Figure 2: the broad band at 3,293 cm^–1^ (Amide A) is attributed to hydrogen bonding coupled with NH-stretching; the peak at 1,651 cm^–1^ (Amide I) is attributed to C-O stretching/hydrogen bonding coupled with COO-; the peak at 1542.94 cm^–1^ (Amide II) is attributed to NH bending coupled with CN stretching (42).

These peaks underwent various changes after binding of HES to WP: the amide A peak shifted to higher wavenumbers; the amide I peak moved to lower wavenumbers; and the amide II shifted from 1542.94 to 1545.86 cm^–1^. Moreover, there was a decrease in the intensities of all these peaks. These changes may be due to a hydrophobic interaction between the oxygen atom and hydroxyl group of HES and the C = O and C-N groups of the WP (43, 44). Researchers reported a similar finding when using FTIR spectroscopy to study the interaction of gelatin with tannic acid and grape seed proanthocyanidins (17).

The amide I band is more sensitive to changes in the secondary structure of proteins than the amide H band (17). Thus, the amide I spectra were deconvoluted to quantitatively analyze the secondary structure of the proteins before and after binding of the HES (Table 3). Before binding, the secondary structure of WP consisted of 32.4% of β-sheet, 26.7% of β-turn, 22.1% of a-helix, and 18.9% of random coil. After binding to HES, there was a major decrease in the α-helix (19.6%) and β-sheet (27.2%) content of the WP, and an increase in the β-turn and random coil structure. These changes suggest that binding of HES to the protein caused the ordered regions of the polypeptide chain to partially unfold. This result is consistent with previous studies, which reported a similar change in secondary structure in casein after interacting with tea polyphenols (42).

**TABLE 3:** Secondary structure contents in WP and WP-HES.

Samples	α-helix	β-sheet	β-turn	Random coil
WP	22.08%±0.56^a^	32.35%±0.32^a^	26.71%±0.21^b^	18.86%±0.14^b^
WP-HES	19.55%±0.38^b^	27.16%±0.46^a^	32.05%±0.28^a^	21.24%±0.33^a^

Results are expressed as means ± *SD* (*n* = 3). Values with different uppercase letters in the same column are significantly different (*p* < 0.05).

### 3.7. Molecular docking simulation

**FIGURE 4:**
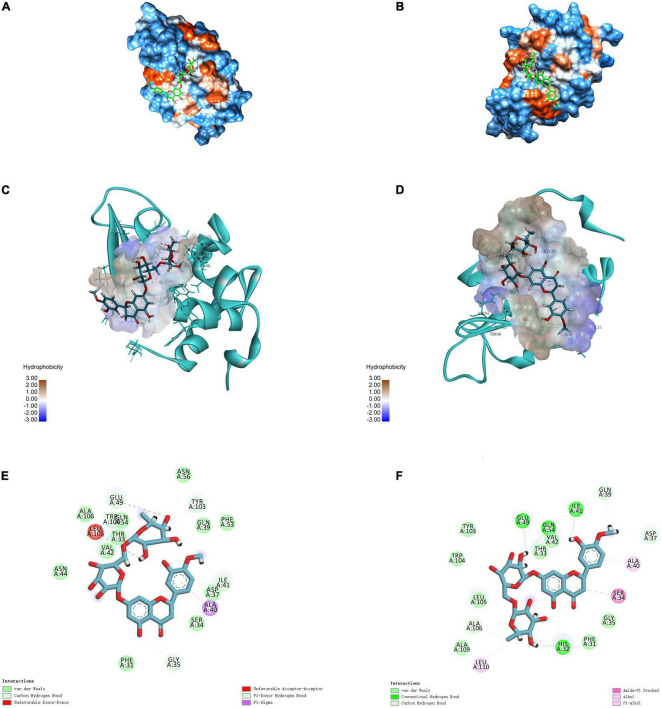
3D diagrams of α-LA-HES **(A)** and β-LG-HES **(B)**; the hydrophobicity surface of α-LA **(C)** and β-LG **(D)** interacted with HES, the red color and blue color represent hydrophobicity and hydrophilicity, respectively; 2D schematic interaction diagram α-LA-HES **(E)** and β-LG-HES **(F)**, the color of amino acid residue is drawn by interaction.

Molecular docking simulation is a useful tool for providing insights into the interaction between receptor and ligand molecules (24, 42). In our simulations, HES was chosen as a ligand, whereas α-LA and β-LG, as the main components in WP, were selected as receptors. The most likely molecular interaction conformations of α-LA/β-LG and HES, which were taken to be the ones with the lowest energy score, are shown in Figure 4. The 3D molecular docking mode (Figure 4A) and 2D interaction diagram (Figure 4E) suggest that HES inserted itself into a hydrophobic cavity in the β-LG. HES was mainly surrounded by 18 amino acid residues on the β-LG, including Gln 39, Asp 37, Ala 40, Ser 34, Gly 35, Phe 31, His 32, Leu 110, Ala 109, Ala 106, Leu 105, Trp 104, TYR 103, Glu 49, Gln 54, Val 42, Thr 33, Ile 41. Among them, five hydrophobic interaction forces (four Pi-alkyl groups and one Amide-Pi Stacked) were found between the aromatic rings of HES and the alkyl groups of Ala 106, Leu 110, His 32, Ser 34, and Ala 40. Additionally, four hydrogen bonds were also observed between the H-acceptor sites of Glu 49, Gln 54, Ile 41, His 32 and the hydroxyl groups of HES. Similarly, HES entered into the hydrophobic inner cavity on α-LA surface and was surrounded by 18 amino acid residues. However, only one hydrophobic interaction (Pi-Sigma) was found between HES and Ala 40, and van der Waals forces and H-bonding drove the interaction. The different binding forces between α-LA/β-LG with HES could be assigned to their different structures. Obviously, the model of β-LG was more suitable to simulate the interaction between WP and HES, after comparing with the results of thermodynamic and FTIR analysis discussed earlier. The important role of hydrophobic interactions between proteins and phenolics were also reported by other researchers. For instance, WP-mulberry anthocyanin complexes were mainly held together by hydrophobic interactions, with van der Waals forces and H-bonding also making some contribution (43). Similarly, rice glutelin-procyanidin complexes were also found to be mainly held together by hydrophobic attractive forces (20).

### 3.8. Determination of antioxidant activity

**FIGURE 5:**
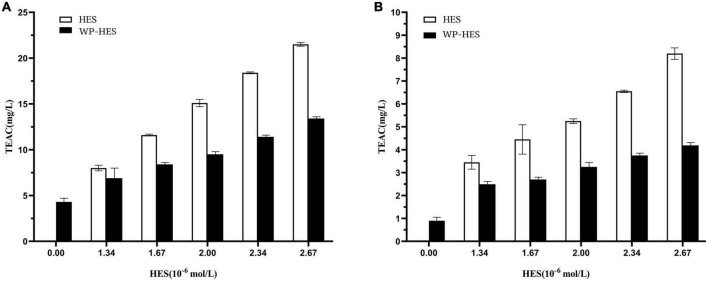
Diphenyl-2-picrylhydrazyl free radical scavenging activity **(A)** and ferric-reducing antioxidant power **(B)** of WP-HES complexes.

The antioxidant capacities of the WP-HES complexes, WPs, and HES were compared using the FRAP reducing power and DPPH radical scavenging assays (34). Although these methods differ in reaction conditions and mechanisms, the antioxidant capacity of all the samples followed similar trends. Both FRAP (Figure 5B) and DPPH (Figure 5A) assays showed that the antioxidant activities of the WP-HES complexes and free HES increased in a dose dependent manner with increasing HES concentration. However, the antioxidant capacity of the WP-HES complexes was significantly lower than that of free HES at the same HES concentration, and higher than that of the WP control (0 μM HES).

As shown in Figure 5A, the DPPH scavenging activity of the WP control was 1.09 mg TE/L solution. This value gradually increased to 4.2 mg TE/L when the HES concentration was raised to 26.7 μM. This result confirmed that the binding of HES to the WP increased the antioxidative activity of the protein. This result agrees with previous reports. For instance, the radical scavenging activity of β-lactoglobulin, α-lactalbumin, and BSA all increased after they bound epigallocatechin gallate (34) whereas the antioxidant activity of gelatin increased after it bound tannic acid (17). Mechanistically, the DPPH assay mainly depends on hydrogen atom donation by antioxidants, which neutralize the DPPH radicals (45, 46). Thus, the DPPH scavenging activity of an antioxidant is mainly due to its hydrogen-donating ability. It is possible that the binding of HES to WP resulted in the loss of phenolic hydroxyl groups in the HES, thereby decreasing its hydrogen donor ability (47). Furthermore, it is known that HES is an antioxidant that contains phenolic hydroxyl groups on its aromatic rings. After binding to the protein, there may still be some phenolic hydroxyl groups exposed in the WP-HES complex, which give it some antioxidant activity.

The principle of the FRAP assay is the reduction of the ferric complex in TPTZ (Fe^3+^-TPTZ) to the ferrous form (Fe^2+^-TPTZ) (20). Therefore, the FRAP assay is mainly measuring the ferric reducing potential of an antioxidant. Figure 5B shows that the FRAP activity of the WP increased from 4.12 to 13.48 mg TE/L after binding the HES. However, the FRAP activity values for all the WP-HES complexes were lower than those of the free HES at the same HES concentration. These results are therefore in agreement with those of the DPPH radical scavenging activity assay. Thus, both methods confirm that binding of HES to WP improved the antioxidant capacity of the protein.

### 3.9. Stability study

In this section, we examined the impact of HES on the resistance of the whey-protein coated droplets in oil-in-water emulsions to changes in pH, salt, and lipid oxidation.

#### 3.9.1. pH stability of emulsions

**FIGURE 6:**
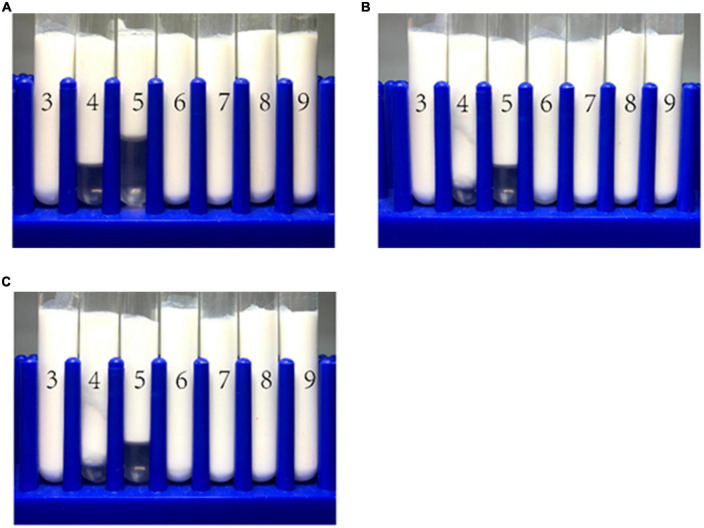
The visual appearance of WP-based emulsion **(A)**, WP-HES (1 mM) based emulsion **(B)**, and WP-HES (2 mM) based emulsion **(C)** under different pH values (numbers on the tubes).

**FIGURE 7:**
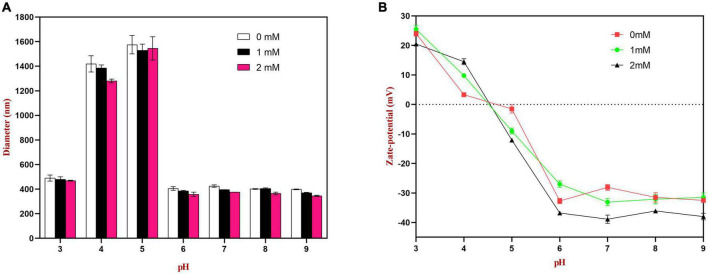
Effects of pH on the particle size **(A)** and zeta potential **(B)** of WP or WP-HES complex stabilized emulsions.

The emulsions used in different food and beverage products must function over a range of pH values, which may affect the stability and physicochemical properties of emulsions. The influence of pH on the particle size, zeta-potential, and microstructure of emulsions stabilized by WPI-HES or WP was therefore measured. The emulsions had a uniform white appearance at pH 3 and from pH 6 to 9, but visibly separated at pH 4 and 5 (Figure 6). The mean particle diameters of the emulsions at pH 4 and 5 were significantly higher than those at other pH values, indicating appreciable droplet aggregation had occurred (Figure 7). Emulsions containing WP-coated oil droplets are predominantly stabilized by electrostatic repulsive interactions. The droplets aggregate at pH values near the isoelectric point of the adsorbed proteins (pH 5) because of the reduction in their electrical charge, which reduces the electrostatic repulsive forces. As a result, they tend to aggregate through van der Waals and hydrophobic attractive forces. Interestingly, the addition of HES reduced the amount of creaming observed in the emulsions. Moreover, the mean particle diameters of the emulsion containing HES were smaller than the corresponding controls, with the particle size decreasing with rising HES concentration. This phenomenon may be caused by non-covalent interactions between the protein and polyphenol molecules.

The spectroscopic analysis and molecular docking simulations discussed earlier showed that the binding interactions between the proteins and polyphenols were mainly a result of hydrophobic bonding, followed by some hydrogen bonding. The presence of the polyphenols may therefore have changed the surface chemistry of the protein-coated oil droplets, thereby altering the interactions between them. Moreover, the binding of the polyphenols caused some partial unfolding of the WP molecules, which may have improved their emulsifying characteristics, leading to the formation of small oil droplets during homogenization. Similar results were also reported by other researchers. Li et al. reported that rice bran protein-catechin complexes formed emulsions with smaller particle sizes than rice bran protein alone (48, 49) reported that soy protein-tea polyphenol complexes led to better emulsion formation and stability than using soy protein alone. It is known that there are many hydrophilic hydroxyl groups in HES. The binding of HES to WP would introduce some phenolic hydroxyl groups onto the surface of the protein molecules, thereby increasing their surface hydrophilicity, which may have reduced the tendency for droplets to aggregation through hydrophobic attraction.

The zeta potential values of the droplets were also measured to provide further insights into the pH sensitivity of the emulsions. According to DLVO theory, higher absolute zeta potential values lead to stronger electrostatic repulsion between droplets, thereby leading to greater resistance to aggregation (3). As shown in Figure 7, at pH 4.0 and 5.0, the surface charge of the emulsion without HES was close to neutral (ζ = + 2.9 and −1.5 mV, respectively). Thus, the zeta potential measurements provide strong support that the poor aggregation stability of the droplets at these pH values was due to a reduction in electrostatic repulsion. The absolute values of the zeta potential at pH 4.0 and 5.0 increased as the HES concentration was increased, indicating that the WP-HES complexes may have increased the resistance of the emulsions to aggregation by increasing the electrostatic repulsion between them. In addition, the binding of the polyphenols to the protein surfaces may have decreased the hydrophobic attraction between the droplets by covering exposed non-polar patches on the protein surfaces. Moreover, the presence of the polyphenols may have increased the steric repulsion between the droplets by increasing the thickness of the interfacial layer. Overall, the particle size and zeta-potential measurements are therefore consistent with the visual appearances and microstructures of the emulsions. Interestingly, the addition of HES caused a slight increase in the pH where zero charge was observed, as well as in the magnitude of the negative charge observed under alkaline conditions. This phenomenon suggests that the combination of HES with WP may have shifted the isoelectric point of the adsorbed proteins to a slightly higher value. Possibly, some of the free hydroxyl groups present in the WP-HES complexes became deprotonated under alkaline conditions, which produced more negative charges (50).

**FIGURE 8:**
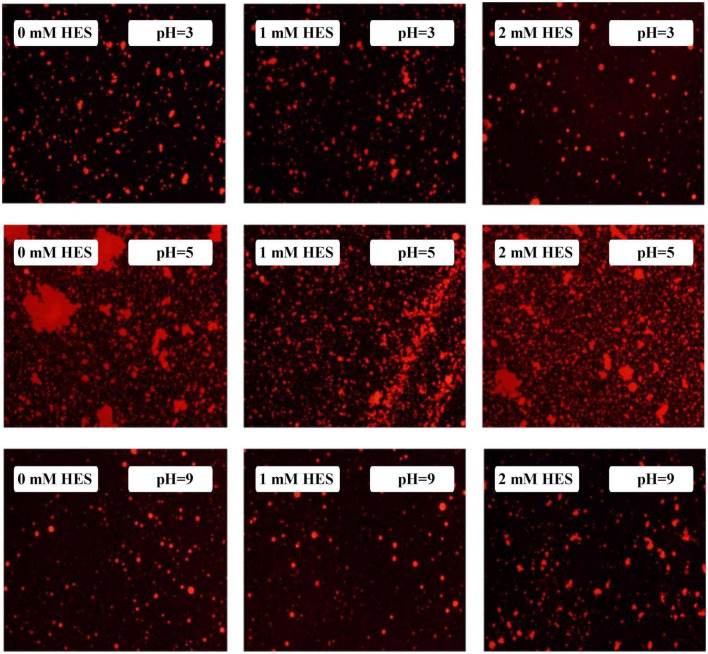
The microstructure of WP based emulsion or WP-HES based emulsions at different pH values.

The microstructures of the emulsions at pH 3, 5, and 9 were examined using an inverted fluorescence microscope (Figure 8). At pH 3, the microscopy images show that both emulsions contained well dispersed small particles. However, there were a few large particles in the emulsions containing WP-coated oil droplets, which were not seen in the ones containing WP-HES-coated droplets. At pH 5, both emulsions contained large aggregates, but the individual aggregates were much larger in the WP-emulsions than in the WP-HES ones. This result suggests that HES could partially inhibit the aggregation of the protein-coated droplets. At pH 9.0, uniformly distributed small particles were observed in both emulsions, with only a few small aggregates being seen, which can be attributed to the strong electrostatic repulsion between the highly negatively charged droplets under alkaline conditions. These results are therefore consistent with the appearance and particle size analysis of the emulsions.

#### 3.9.2. Salt stability

Food emulsions may be used in food and beverage products with different ionic compositions, and so it is important to ascertain the resistance of droplets to increases in high ionic strength (51). The effect of salt concentration on the stability of WP- and WP-HES-emulsions after being stored at pH 3, 7, and 9 overnight was investigated.

**FIGURE 9:**
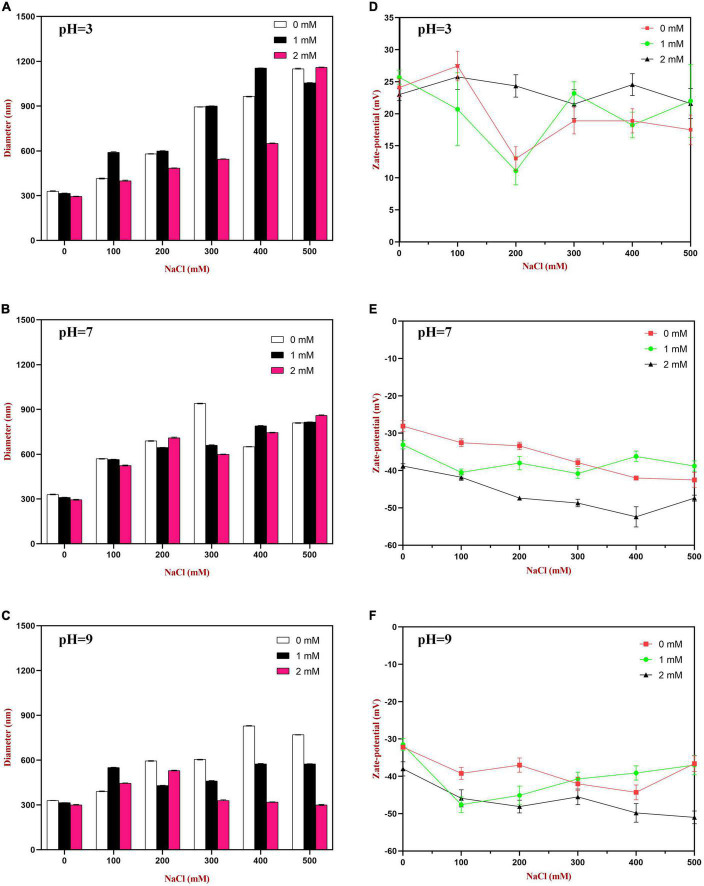
The particle size **(A–C)** and zeta-potential **(D–F)** changes of WP based emulsion or WP-HES based emulsions under different pH values and salt concentrations.

**FIGURE 10:**
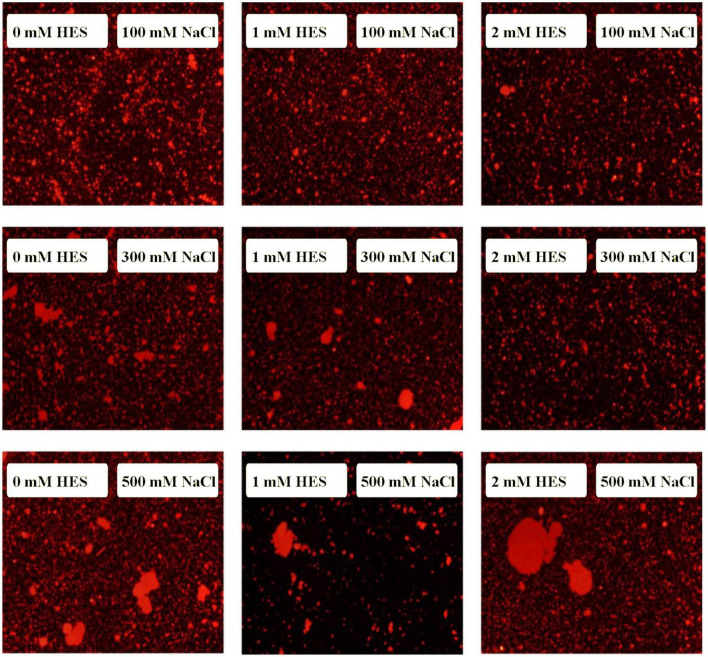
The microstructure of emulsions with different salt ion concentrations under acidic condition.

At pH 3, the mean particle diameter of all the emulsions increased in a dose-dependent manner with increasing NaCl concentration (Figure 9). This increase in particle size can be attributed to electrostatic screening effects. Emulsions containing protein-coated oil droplets are mainly stabilized by electrostatic repulsion. The addition of mineral ions screened these electrostatic repulsive forces, which can promoted aggregation due to hydrophobic and van der Waals attractive forces (52). Compared to the control emulsions, the WP-HES-emulsions containing 2 mM HES had significantly smaller particle sizes (except at 500 mM NaCl). The electrophoresis measurements showed that all the emulsions contained positively charged droplets and that the magnitude of the zeta potential decreased with increasing salt concentration (Figure 9D), which can again be attributed to electrostatic screening effects. Microscopy analysis also confirmed that aggregation occurred as the NaCl concentration increased for both WP-HES- and WP-emulsions (Figure 10). The higher salt-stability of the emulsions containing the higher level of HES may be due to a reduction in hydrophobic attraction and increase in steric repulsion between the droplets (51, 53).

At pH 7, the particle size of all the emulsions increased with increasing NaCl concentration, which can again be attributed to screening of electrostatic repulsive forces. In this case, however, the presence of HES did not improve emulsion stability. Surprisingly, the magnitude of the zeta-potential actually increased slightly with increasing salt concentration (Figure 9E). The addition of 2 mM HES led to a slight increase in the magnitude of the negative charge on the oil droplets, which may have been due to deprotonation of the polyphenol hydroxyl groups (17).

At pH 9, the addition of salt also promoted some droplet aggregation as seen in the particle size and microstructure images (Figure 9). This effect can again be attributed to screening of the electrostatic repulsion between the droplets by salt ions. In this case, the presence of HES improved the salt-stability of the emulsions, with a smaller rise in mean particle diameter and fewer larger particles in the microscopy images. This may have been at least partly because incorporating HES into the emulsions increased the magnitude of the negative zeta potential of the droplets, thereby increasing the electrostatic repulsion (54). The greater effect at pH 9 than at pH 7 may have been because a higher fraction of phenolic hydroxyl groups was deprotonated under stronger alkaline conditions.

#### 3.9.3. Measurement of lipid oxidation

Oxidative degradation of lipids is one of most important factors limiting the shelf life and acceptability of many fatty foods (55). The secondary reaction products formed lead to rancidity and potential toxicity (17). In our experiments, the coix seed oil used to formulate the emulsions is rich in unsaturated fatty acids and is therefore highly susceptible to oxidation. The oil droplets in the emulsions prepared in our study were coated with WP molecules, which may offer some protection against oxidation. In this section, we examined the impact of HES on their antioxidant properties.

##### 3.9.3.1. PV measurements

**FIGURE 11:**
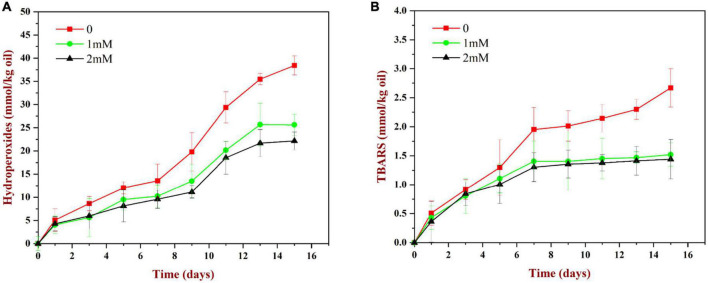
Change in PV **(A)** and TBARS **(B)** values for WP based emulsion or WP-HES based emulsions during storage at 55°C for 15 days.

As shown in Figure 11A, the content of lipid hydroperoxides (primary reaction products) in all samples increased gradually during storage. The peroxide value increased relatively slowly during the first 7 days, but then increased rapidly. Compared to the WP-emulsions, the PV values of the WP-HES-emulsions were significantly reduced. Moreover, the extent of lipid oxidation decreased in a dose dependent manner as the HES concentration was increased. This effect may be because the binding of polyphenols to the WP molecules enhanced their antioxidant activity (Figure 5). Similar results have also been reported for other polyphenol-protein complex stabilized emulsions (17, 35).

##### 3.9.3.2. TBARS measurements

As shown in Figure 11B, the TBARS values (secondary reaction products) increased during storage, which indicated that some of the primary oxidation products had broken down. The TBARS values increased steadily during the first 7 days of storage and then remained constant or only increased slowly. The addition of HES (1 or 2 mM) to the emulsions reduced the concentration of TBARS formed at longer storage times. These results suggest that the polyphenols may have partially suppressed the formation of secondary reaction products, again possibly by binding to the adsorbed proteins and forming an interfacial layer with strong antioxidant properties.

## 4. Conclusion

In summary, this study has provided evidence for the hypothesis that non-covalent interactions are formed between HES and WP leading to the formation of WP-HES complexes. Molecular dynamic simulations and spectroscopy analysis suggested that hydrophobic interactions were the most important forces holding these complexes together, but that hydrogen bonding also played a role. Spectroscopy analysis also showed there was a change in the secondary structure of the WP molecules after binding the polyphenol, with decreases in α-helix and β-sheet structures and increases in β-turn and random coil structures. The binding of the HES to the WPs enhanced their antioxidant activity, as well as their ability to increase the resistance of emulsions to changes in pH and salt concentration. Overall, our results suggest that WP-HES complexes may be used in the food industry as antioxidant emulsifiers that have better performance than WP alone.

## Data availability statement

The original contributions presented in this study are included in the article/supplementary material, further inquiries can be directed to the corresponding authors.

## Author contributions

YW: conceptualization, software, formal analysis, investigation, and writing—review and editing. YG: conceptualization, methodology, formal analysis, and investigation. LTZ: resources and visualization. MY: investigation. LZ: software, resources, and funding acquisition. CB: supervision, funding acquisition, data curation, and project administration. DM: validation and supervision. All authors contributed to the article and approved the submitted version.

## Footnotes

1
http://PubChem.ncbi.nlm.nih.gov

2
http://www.rcsb.org
